# The Species-Specific Responses of Freshwater Diatoms to Elevated Temperatures Are Affected by Interspecific Interactions

**DOI:** 10.3390/microorganisms6030082

**Published:** 2018-08-07

**Authors:** Yun Zhang, Chengrong Peng, Zhicong Wang, Jinli Zhang, Lijie Li, Shun Huang, Dunhai Li

**Affiliations:** 1 Key Laboratory of Algal Biology, Institute of Hydrobiology, Chinese Academy of Sciences, Wuhan 430072, China; zhangy@ihb.ac.cn (Y.Z.); pengcr@ihb.ac.cn (C.P.); wangzc@ihb.ac.cn (Z.W.); zhangjl@ihb.ac.cn (J.Z.); lilj@ihb.ac.cn (L.L.); huangshun@ihb.ac.cn (S.H.); 2 University of Chinese Academy of Sciences, Beijing 100049, China

**Keywords:** diatoms, interspecific interactions, seasonal succession, warming scenarios

## Abstract

Numerous experimental simulations with different warming scenarios have been conducted to predict how algae will respond to warming, but their conclusions are sometimes contradictory to each other. This might be due to a failure to consider interspecific interactions. In this study, the dominant diatom species in a seasonal succession were isolated and verified to adapt to different temperature ranges by constant temperature experiment. Both unialgal and mixed cultures were exposed to two fluctuant temperature treatments that simulated the temperature variations from early spring to summer, with one treatment 4 °C higher (warming scenario) than the other. We found that the specific response of diatoms to warming was affected by interspecific interactions. Spring warming had no significant effect on eurythermal species and had a positive effect on the abundance of warm-adapted diatom species, but interspecific interactions reduced this promotional effect. Cold-adapted species had a negative response to spring warming in the presence of other diatom species but had a positive response to early spring warming in the absence of interspecific interactions. In addition, warming resulted in the growth of all diatom species peaking earlier in unialgal cultures, but this effect could be weakened or amplified by interspecies interactions in mixed cultures. Our results suggest that the specific diatom species with different optimal growth temperature ranges responding to warming were expected if there were no interspecific interactions. However, in natural environments, the inevitable and complex interspecific interactions will influence the responses of diatoms to warming. This important factor should not be ignored in the prediction of organism responses to climate warming.

## 1. Introduction

Diatoms (Bacillariophyceae) are generally considered to be better adapted to lower temperatures than are other algae [1]. They are usually prevalent in spring in subtropical water bodies [2,3,4,5]. Relatively low temperatures in spring are suitable for the growth of many diatom species [6,7]. Some diatom species even form serious blooms in spring and cause a series of ecological problems, such as low water transparency, bad odors, clogging or sedimentation in water treatment processes, and a decrease in the perceived aesthetic value of the water body [8].

Climate change has a great influence on aquatic habitats, with most of the potential effects being specifically related to global warming [9]. Global warming is likely to result in an increased water temperature in aquatic ecosystems, especially in winter and spring [10], a consequence of a warmer and shorter winter is an earlier onset of spring. For aquatic systems, the phytoplankton spring bloom characterizes the end of winter. The spring bloom is the first step in the Plankton Ecology Group (PEG) model of phytoplankton succession [11]. Ample nutrient availability, increasing day length, and rising water temperatures stimulate blooms of fast-growing diatoms. Warming will change the temporal rhythm and intensity of aquatic events in spring [12], for example, it may increase the probability of warm-adapted diatoms occurring in blooms [13,14,15] and advance the formation of blooms [16,17,18] in spring. These events are not conducive to the effective management and protection of water bodies, especially drinking-water reservoirs, which are usually dominated by diatoms [19]. Drinking-water reservoirs play an increasingly important role in water supply, and the water quality in these reservoirs is closely related to human health [20]. The blooms in drinking-water reservoirs not only destroy the structure of aquatic ecosystems, but more importantly, they produce odorous substances and decrease the water quality, which seriously affects the safety of the water supply.

Seasonal succession among different diatom species is also a common ecological phenomenon in drinking-water reservoirs [21], and this may be related to the optimum temperature for the growth of specific diatoms being different [22]. Diatom species that favor different temperature ranges may respond to warming differently [23]. Eurythermal species can grow well over a relatively wide temperature range, and are common in all seasons. Therefore, these eurythermal species may be dominant during periods of long-term climate change [23]. Cold adapted species have a competitive advantage in cold water and could be replaced by warm adapted species if the temperature increases due to global warming [24]. However, the complexity of the natural environment has prevented any firm conclusions being reached about the specific effects of warming on diatoms. Many studies have focused on how to use phytoplankton biomass to reflect global warming, but their conclusions are different and even contradictory [25,26,27,28]. An increase in temperature can improve the metabolic rate and growth rate of organisms as well as influence trophic interactions [29,30], but it is still not clear what causes the aforementioned divergence. Striebel et al. (2016) suggested three potential sources of divergence: abiotic conditions, species composition, and temperature disturbance [31]. However, another potentially important factor, interspecific interactions, has rarely been considered in studies of the phytoplankton response to climate warming. In studies where interspecific interactions occur, investigators may deviate in the effects of warming on algae. For example, under warming the phytoplankton community structure changes, but it is unclear whether these changes are caused directly by warming or indirectly by interspecific interactions.

Both models and manipulative experiments have indicated that interspecific interactions could affect the responses of species to warming [32,33]. Interspecific interactions may directly affect the growth of species. Competition for resources (nutrients, space, and light) is the most common interactions between species, and weak competitors for those resources often end up being eliminated due to growth constraints [34]. Unfavorable conditions for algal species may also arise if they interact with each other via secondary metabolites that affect cell physiology and growth of their competitors [35]. In addition, interspecific interactions may themselves be affected by climate warming, in terms of both their pattern and intensity [36], which would further influence the response of algae to climate warming. For example, climate warming increases the toxicity and abundance of harmful diatom blooms in California coastal waters [37], which leads to a more intensive inhibitory effect on other organisms. Some species even display negative competitive interactions in cold climates and positive promotional interactions in warm climates [38].

There are currently many reports of the impacts of warming on blue-green algae and the whole phytoplankton community, but there have been few studies of specific diatom species, even though diatoms are the dominant community in most drinking-water reservoirs. Therefore, we isolated five dominant diatom species from a drinking water reservoir in different seasons and explored the effects of elevated temperature (+4 °C, in line with warming scenarios predicted for temperate latitudes by the end of the 21st century) [39] on unialgal and mixed cultures. Before the elevated temperature experiment, we cultured five diatom species at six constant temperatures to determine whether they were adapted to different temperature ranges. The aim of the study was to test our hypothesis that diatoms with obvious seasonal succession adapted to different temperature ranges, and if their specific responses to warming are impacted by interspecific interactions.

## 2. Material and Methods

### 2.1. Diatom Isolation

Five diatom species, *Fragilaria nanana* Lange-Bertalot, *Achnanthidium catenatum* (Bily and Marvan) Lange-Bertalot, *Aulacoseira ambigua* (Grunow) Simonsen, *Ulnaria ulna* (Nitzsch) Compère and *Asterionella formosa* Hassall, were isolated from a drinking-water reservoir (E114°32′–114°35′, N31°17′–31°23′), the Jinshahe Reservoir, in Hubei province, China. The abundance of the five diatom species presented an obvious seasonal succession (the variation of their abundance is shown in Figure S1). Some of the biological and ecological characteristics of the five diatom species are listed in Table 1. Morphological identification of the five diatom species was carried out by light microscopy (Olympus, Tokyo, Japan) at 1000× magnification (oil immersion lens). For molecular biology identification, the DNA of the five diatom species was extracted, and a conserved region of 18S ribosomal RNA was amplified and sequenced, and the sequences were subjected to nucleotide BLAST comparison [40]. The five diatom strains were maintained with Csi medium (Freshwater Algae Culture Collection at the Institute of Hydrobiology, http://algae.ihb.ac.cn/Products/ProductDetail.aspx?product=15) in unialgal cultures.

### 2.2. Growth under Constant Temperature

Unialgal cultures of diatoms were grown in illuminated incubators at six constant temperatures (10 °C, 15 °C, 20 °C, 25 °C, 30 °C, and 35 °C), based on the temperature we surveyed, which varied annually from 6 °C to 35 °C. The cultures were subjected to a 12:12 light:dark (L:D) cycle, with an intensity of 60 μmol photons m^−2^·s^−1^ provided by cool-white fluorescent lamps (Shanghai, China). Each 300 mL polycarbonate flask contained 200 mL Csi medium with an initial density of about 1.0 × 10^6^ cells·L^−1^. Three replicates of each diatom species were cultured at each temperature. Flasks were shaken manually twice every day to keep the cells in suspension. Cell density, chlorophyll *a* (chl-*a*) concentration, and chlorophyll fluorescence were determined every 3 days. The experiments lasted 25 days.

### 2.3. Experimental Design of the Simulated Warming Scenario from Early Spring to Summer

**Figure 1:**
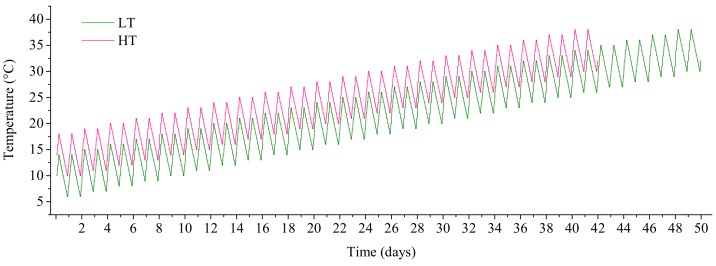
The simulated temperatures for the duration of the experiment for low temperature (LT) and high temperature (HT) (+4 °C) treatments.

Simulated temperatures fluctuated in our study, but it was a regular fluctuation under ideal conditions, and not as complex as the field. The simulated temperature fluctuations were achieved in two temperature-controlled light incubators. The temperature was changed every 2 h according to the actual temperature changes during a day, and therefore 12 temperature levels were set daily. Every 2 days, the temperature of each level was increased by 1 °C in both light incubators. The temperature in one of the incubators was 4 °C higher (high temperature, HT) than the other (low temperature, LT) throughout the experiments. The temperature changes in the two incubators are shown in Table 2 and Figure 1. The five diatom species were cultured in two modes of unialgal and mixed cultures, using 300 mL polycarbonate flasks in the two incubators. To eliminate the influence of nutrient depletion on the growth restriction of diatoms, fresh medium with high nutrient concentration of equal volume to the sampled culture was added to the flasks every 2 days. The volume of the sampled culture was small (4 mL) and the dilution was therefore negligible. Cell density, chlorophyll fluorescence and the concentration of dissolved silicate (Figure S2) in the medium were determined every 2 days. Diatom cells were counted with a counting chamber (0.1 mL) under a light microscope (Olympus). Cell counts for different diatom species in mixed cultures were performed. The biovolume of diatom species was calculated according to [42]. The biomass (wet weight) of each taxonomic group was derived based on an assumption of a plasma density of 1 g/cm^3^ across all taxa [43]. The experiment lasted until the photosynthetic parameter values (*Fv*/*Fm*) of algal cells dropped to near zero because of the high temperature.

### 2.4. Growth Evaluation

The specific growth rate *μ* (day^−1^) of each strain in the exponential phase was calculated by applying:

*μ* = (ln *N_t_*_2_ − ln *N_t_*_1_)/∆*t*

where *N_t_*_1_ is the initial cell density, *N_t_*_2_ is the cell density at the next observation, and ∆*t* (days) is the interval between observations.

Based on the growth trends of diatoms in the simulated warming scenario experiment, their growth could be artificially divided into three phases. The first phase was an initial and exponential growth period, in which there was rapid growth of all diatom species (phase 1: day 1–10 for the mixed culture and day 1–15 for the unialgal culture). The second phase was a stable period (phase 2: day 11–30 for the mixed culture and day 16–30 for the unialgal culture). The final phase was a decline period for all diatom species (phase 3: day 31–50 for LT and day 31–42 for HT). The specific growth rates were calculated accordingly.

### 2.5. Chlorophyll Fluorescence Measurement

The maximal yield of photochemical energy conversion (*Fv*/*Fm*) was measured using a Water-PAM Chlorophyll Fluorometer (Walz, Effeltrich, Germany).

### 2.6. Statistical Analysis

A normality test of all data was conducted by a Shapiro–Wilk test and homogeneity test of the variance for normally data was conducted by Bartlett’s test. Differences in growth rates and cell densities at six temperatures were compared using a one-way ANOVA followed by a Tukey HSD post-hoc test if the data was normally distributed and had equal variances; if not, a nonparametric Kruskal–Wallis test was conducted, and post-hoc all-pairwise Connover–lnman test. The difference in the cell densities between LT and HT was tested using a student’s t test if the data complied with a normal distribution; if not, a Kolmogorov–Smirnov test was used. Statistical analyses were performed using R 3.4.1 software. Graphs were generated in Origin 9.0.

## 3. Results

### 3.1. The Growth of Diatoms under Constant Temperature

**Figure 2:**
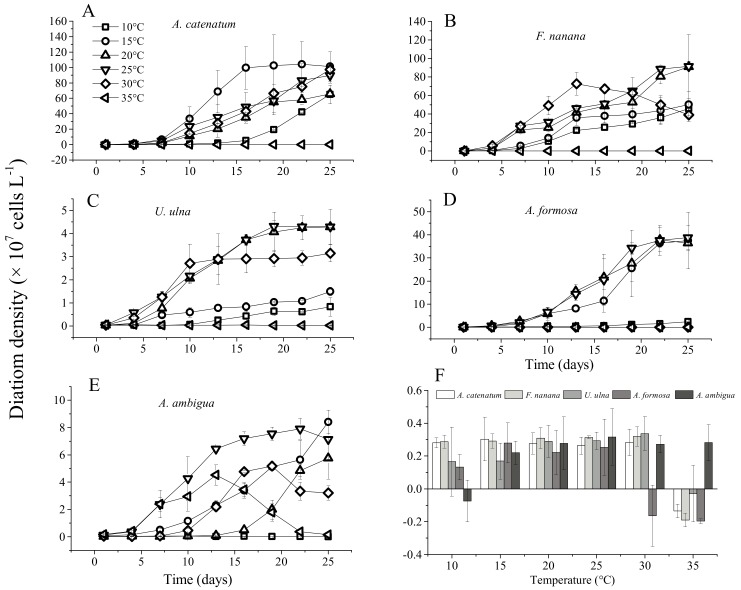
Growth curves and specific growth rates of the five diatom species at six constant temperature levels. (**A**) *Achnanthidium catenatum*; (**B**) *Fragilaria nanana*; (**C**) *Ulnaria ulna*; (**D**) *Asterionella formosa*; (**E**) *Aulacoseira ambigua*; (**F**) The specific growth rates of the five diatom species.

**Figure 3:**
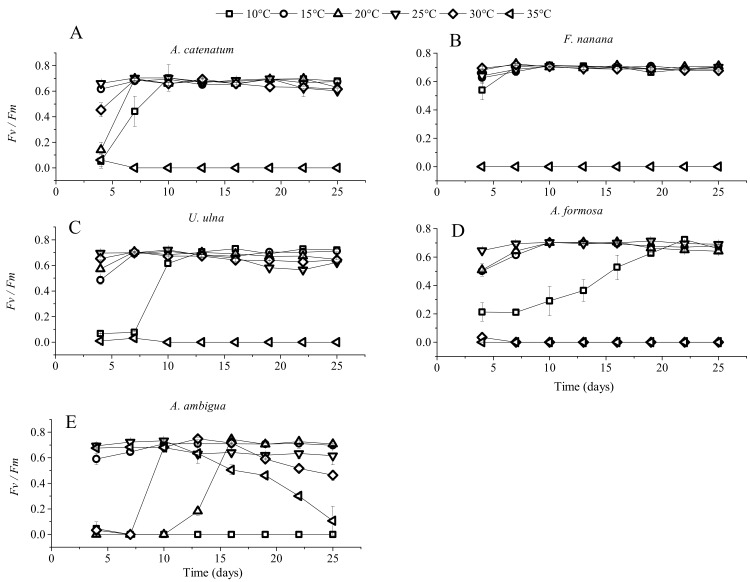
Effective quantum yields of PS II (*Fv*/*Fm*) of the five diatom species at six constant temperatures. (**A**) *Achnanthidium catenatum*; (**B**) *Fragilaria nanana*; (**C**) *Ulnaria ulna*; (**D**) *Asterionella formosa*; (**E**) *Aulacoseira ambigua*.

*A. catenatum* and *F. nanana* had high growth rates from 10 °C to 30 °C, and their growth rates were not significantly different among these five temperatures (Figure 2). The two diatom species could not survive at 35 °C, and their *Fv*/*Fm* values dropped to zero during the incubation period (Figure 3). The growth rate of *U. ulna* at 10 °C and 15 °C was significantly lower (*p* < 0.05) than at 20 °C, 25 °C, and 30 °C. *A. formosa* could not grow when the temperature reached 30 °C. Its growth rate was highest at 15 °C. *A. ambigua* was the only species that could grow at 35 °C, but its cell densities and *Fv*/*Fm* values decreased rapidly after 13 days of culture. At 30 °C, the *Fv*/*Fm* value of *A. ambigua* decreased on day 19 and cell density decreased on day 22. *A. ambigua* had a negative growth rate at 10 °C.

**Figure 4:**
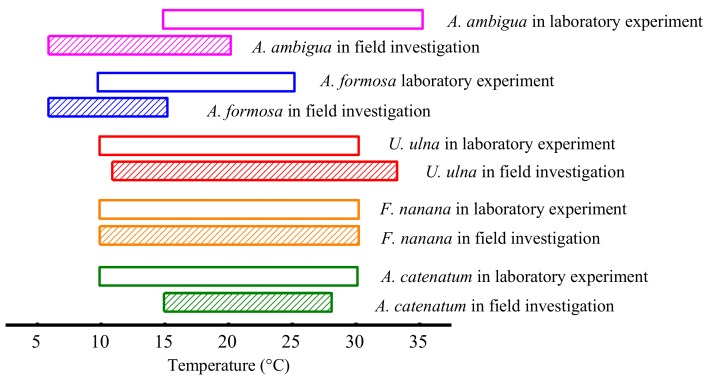
Temperature ranges of the diatom species observed in field studies (cross-hatch) and in laboratory experiments where growth was recorded (open bars). Magenta: *Aulacoseira ambigua*; Blue: *Asterionella formosa*; Red: *Ulnaria ulna*; Orange: *Fragilaria nanana*; Olive: *Achnanthidium catenatum*.

Based on the growth experiment with diatoms under a constant temperature, both *F. nanana* and *U. ulna* adapted to the same temperature range as that identified in the field investigation (Figure 4). *A. catenatum* adapted to a wider growth temperature range in the laboratory experiment as compared to the temperature range obtained from field investigation. *A. ambigua* and *A. formosa* exhibited higher temperature ranges in the laboratory experiments than were found in the field investigations. However, *A. ambigua* had the biggest difference in temperature ranges between the laboratory experiments and the field investigations.

### 3.2. Diatom Growth under Fluctuant Temperatures in Unialgal and Mixed Cultures

**Figure 5:**
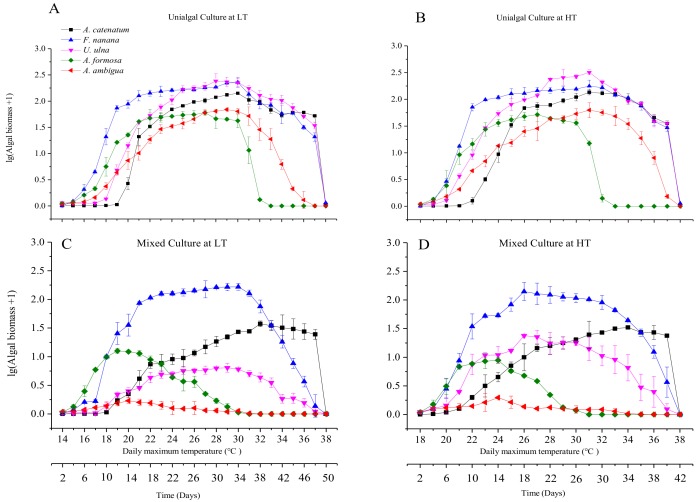
Volumetric biomass of the five diatom species at fluctuant temperatures. The biomass data were lg (x + 1) transformed. (**A**) Biomass (mg/L) of the five diatom species in unialgal cultures at low temperature (LT); (**B**) Biomass of the five diatom species in unialgal cultures at high temperature (HT) (+4 °C); (**C**) Biomass of the five diatom species in mixed cultures at LT; (**D**) Biomass of the five diatom species in mixed cultures at HT.

The experiment in the (+4 °C) HT groups ended 8 days earlier than in the LT groups, and both experiments ended when the highest temperature was 38 °C. The biomass of all diatom species in unialgal cultures were significantly (*p* < 0.05) higher than that in mixed cultures, except for *F. nanana* (Figure 5). In unialgal cultures, all diatom species had an obvious exponential growth phase, with wide peaks. However, in mixed cultures, the growth of some species was visibly inhibited. *A. ambigua* was the species with the most noticeable growth restriction, and its biomass was maintained at a low level. *A. formosa* was the first species to display a peak in growth and the first species in which the biomass decreased. It had a short exponential growth phase and a narrow peak in the early period of the experiment. *U. ulna* had a significantly higher biomass at HT than LT, and its exponential growth phase in mixed cultures was significantly shorter than that in unialgal cultures. *A. catenatum* had a flatter growth phase in mixed cultures than that in unialgal cultures. The growth of *F. nanana* was least affected by co-cultures.

### 3.3. The Effect of Elevated Temperature (+4 °C) on Diatoms

**Figure 6:**
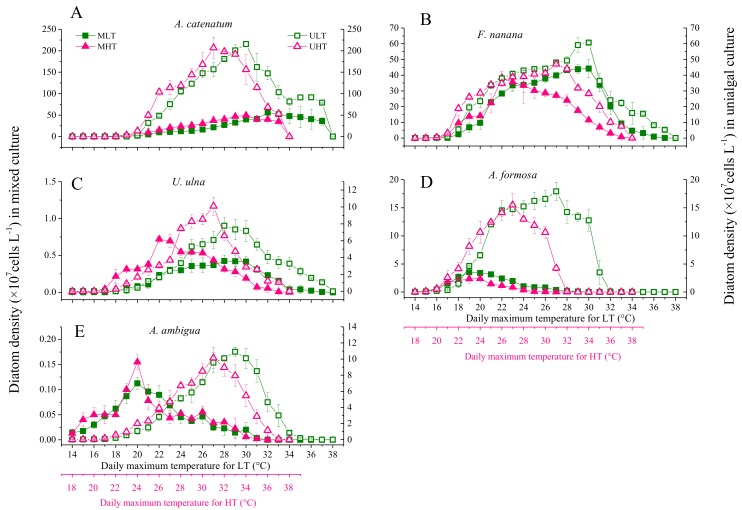
Changes in cell density in unialgal and mixed cultures of five diatom species at fluctuant temperatures. MLT: mixed cultures at low temperature. MHT: mixed cultures at high temperature (+4 °C). ULT: unialgal cultures at low temperature. UHT: unialgal cultures at high temperature (+4 °C). Solid square means MLT, open square means ULT, Solid upward pointing triangle means ULT, open upward pointing triangle means UHT. (**A**) *Achnanthidium catenatum*; (**B**) *Fragilaria nanana*; (**C**) *Ulnaria ulna*; (**D**) *Asterionella formosa*; (**E**) *Aulacoseira ambigua*.

The elevated temperature significantly increased (*p* < 0.05) the cell densities of *A. catenatum* and *U. ulna* within a certain temperature range (Figure 6). The increase in the cell densities in *A. catenatum* at HT occurred when the daily maximum temperature was lower than 32 °C and 34 °C in unialgal and mixed cultures, respectively, and thereafter the opposite pattern occurred. The increase in the cell density of *U. ulna* occurred when the daily maximum temperature was lower than 32 °C in both unialgal and mixed cultures. The temperature elevation did not significantly increase the cell densities of *F. nanana* and *A. ambigua* in the two cultures. However, when the daily maximum temperature exceeded 32 °C, the elevated temperature reduced the cell densities of *F. nanana* and *A. ambigua* in unialgal cultures. In mixed cultures, the decrease in *F. nanana* cell density caused by warming occurred when the daily maximum temperature reached 28 °C, but there was no decrease in *A. ambigua* cell density caused by warming throughout the experiment. In the early period of the experiment, when the temperature was <24 °C, the cell density of *A. formosa* at HT in the unialgal cultures was higher than that at LT, but in the mixed cultures, the elevated temperature did not increase cell density. *A. formosa* was the earliest species (26 °C in unialgal cultures and 22 °C in mixed cultures) in which the cell density at HT was significantly lower than that at LT.

**Figure 7:**
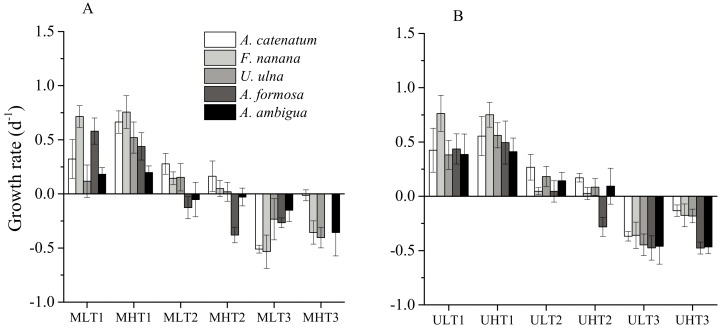
The specific growth rates of five diatom species in three phases of growth at fluctuant temperatures. Phase 1 (day 1–10 for the mixed culture and day 1–15 for the unialgal culture) at both LT and HT; Phase 2 (day 11–30 for the mixed culture and day 16–30 for the unialgal culture) at both LT and HT; Phase 3 (day 31–50 for LT and day 31–42 for HT in both mixed and unialgal cultures). (**A**) The specific growth rates of diatoms in mixed cultures. MLT: mixed cultures at low temperature. MHT: mixed cultures at high temperature (+4 °C); (**B**) The specific growth rates of diatoms in unialgal cultures. ULT: unialgal cultures at low temperature. UHT: unialgal cultures at high temperature (+4 °C).

The specific growth rates were calculated in three phases as described in Methods (Figure 7). In phase 1, *A. catenatum* and *U. ulna* in both unialgal and mixed cultures had significantly higher growth rates at HT than at LT (*p* < 0.05). There were no significant differences in the growth rates of *F. nanana* and *A. ambigua* between HT and LT. *A. formosa* had a lower growth rate at HT than at LT in mixed cultures, but a higher growth rate at HT in unialgal cultures. In phase 2, the growth rates of all diatom species at HT were lower than at LT, with *A. formosa* having a significantly lower growth rate at HT than at LT (*p* < 0.05). It was also the only species with a negative growth rate when cultured in unialgal cultures at HT. With the temperature increase, all diatom species showed negative growth in phase 3.

### 3.4. The Differences in Diatom Responses to Warming Scenarios in Unialgal Versus Mixed Cultures

*F. nanana* and *A. ambigua* had the same responses to the warming scenario in unialgal and mixed cultures when warming occurred in the spring scenario (Figure 6); however, warming decreased the cell density of *F. nanana* earlier in mixed cultures than in unialgal cultures when warming occurred in the early summer scenario. *A. ambigua* also responded to the summer warming scenario differently in unialgal cultures than in mixed cultures. *A. catenatum* and *U. ulna* had significantly higher densities under the spring warming scenario in the two culture modes; however, the increases in mixed cultures were significantly smaller (*p* < 0.05) than in unialgal cultures. Under the summer warming scenario, warming decreased the cell density of *A. catenatum* earlier in unialgal cultures than in mixed cultures. The opposite responses to elevated temperature were found for *A. formosa* in the two culture modes. Warming resulted in an earlier peak of cell density for all diatoms in unialgal cultures because of the faster growth, except for *F. nanana*, which peaked earlier because of its earlier decline. However, in mixed cultures the results were different. The cell density peaks of *F. nanana* and *U. ulna* at HT formed much earlier than at LT, but *A. formosa* and *A. ambigua* peaked at the same time in the two temperature groups.

## 4. Discussion

### 4.1. Specific Responses of Diatoms to Warming

The adapted growth temperature ranges of most diatom species in laboratory experiments were similar to or consistent with those observed in the field, with the exception of *A. ambigua* (Table 1, Figure 4 and Figure 6). Our experimental results confirmed that these diatom species adapted to different temperature ranges, which indicated their specific responses to warming when there were no interspecific interactions.

There was a wide range of growth temperatures for *U. ulna* in both the laboratory experiments and field investigation. The laboratory experiments showed that it had a significantly higher specific growth rate from 20 °C to 30 °C than from 10 °C to 15 °C. Cox (1993) also indicated that *U. ulna* responded positively to a temperature increase from 5 °C to 15 °C, and had higher growth rates at 15–25 °C than at 5–15 °C [44]. In our study, the most significant positive response to the simulated warming in the spring scenario was found for the warm-adapted eurythermal species. *U. ulna* has also been reported to be an important species that occurs in warm conditions in the Portneuf River, Idaho [45].

*A. catenatum* was observed to be a spring–autumn member of the diatoms in the reservoir, but in laboratory experiments it had a wider range of growth temperatures. It has been reported that *A. catenatum* is the only planktonic species in the genus *Achnanthidium* [46]. It is considered an invasive tropical species in some regions and is also reported to be a bloom-forming species, with blooms probably associated with climate warming [47,48]. In the Jinshahe reservoir, the site where this strain was isolated, this fast-growing species formed a bloom in the spring of 2014, and its abundance reached 3.28 × 10^8^ cells/L [49]. However, in the preceding decade, this species had not been found in the reservoir [50]. In the present study, the abundance of *A. catenatum* significantly increased in the spring warming scenario. This opportunistic species may therefore favor higher temperatures in spring.

The highest abundance of *A. formosa* appeared in winter, and our study further indicated that it could not adapt to high temperatures exceeding 28 °C. Hayakawa et al. (1994) also showed that *A. formosa* has a growth temperature range of 5–25 °C, with the largest growth rate at 20 °C, and no positive growth at 30 °C in culture flask experiments [51]. *A. formosa* exhibited higher temperature ranges in the laboratory experiments than were investigated in the field. This may be because it is affected by other factors in the field, which restricts its growth even within the suitable temperature range. Therefore, the elevated temperatures in the early stage of the experiment, which corresponded to the field temperature in early spring, also significantly increased the cell density of *A. formosa.* However, it could not adapt to as high temperatures as other diatoms, so its density decreased as temperatures increased (Figure 6). The species composition and abundance of diatoms in the northern Ural region has been altered by the increasing temperature during the ice-free season, and *A. formosa* was found to be one of the most frequent planktonic diatoms following diatom compositional shifts [52]. Therefore, we inferred that warming will increase the abundance of *A. formosa* in early spring, but from spring to summer, warming will lower its abundance.

*F. nanana* had the same growth temperature range in laboratory experiments and in the field investigation (Figure 4). Based on these results, we found that it is a eurythermal species. Warming did not significantly increase the abundance of *F. nanana* in spring. Eurythermal species have similar growth rates over a very wide range of temperatures and a slight increase in temperature will not significantly alter their growth. *F. nanana* is a species that is commonly recorded in oligotrophic lakes [53,54]. The summer warming decreased its cell density because the high temperature exceeded its growth temperature range. The results presented here and the field observations of other authors also suggest that the *Fragilaria* genus is a eurythermal genus capable of surviving over a wide range of temperatures [55,56,57].

*A. ambigua* was the only species whose growth temperature range in laboratory experiments was inconsistent with the field investigation. It was a low temperature adapted species in the field. However, it could tolerate a high temperature (35 °C) under constant temperature conditions in laboratory experiments. Due to the complex impact of various factors on organisms in the field, the growth temperature ranges of organisms determined from field investigations are usually narrower than their actual growth temperature ranges. In our field investigation, the silicate concentration was limited for almost all diatoms from late spring to early summer. The low abundance of *A. ambigua* in summer may also not be caused by the high temperature. Typically, reservoirs are deep, and during the summer a thermocline usually develops, which is not favorable to the growth of the large-celled tychoplankonic *A. ambigua* because it will rapidly sink [58]. In Lago de Pa’tzcuaro, *A. ambigua* is a spring and early summer species, as it is today in many other lakes [59]. In addition, there was a high abundance of *A. ambigua* in winter in the field (the lowest temperature measured was 6 °C), but it could not grow at 10 °C in the constant temperature experiment. We are unable to explain this phenomenon. Temperature controls the rate of enzymatic reactions such as the metabolism, photosynthesis, and respiration of phytoplankton, which determine the basic growth rate [60]. Because *A. ambigua* could adapt to low and high temperatures, i.e., a wide temperature range, it also had no significant response to an elevated temperature in spring.

Climate warming has a great influence on spring diatom species, with one of the most obvious impacts being to cause the peaks of spring diatom species to occur earlier [12,16,61,62]. In Müggelsee, phytoplankton blooms, of which diatoms comprise >80% of the biomass, have occurred ~4.5 weeks earlier since 1988 compared with the preceding decade [63]. In our experiments, all the diatom species in the warming scenario peaked earlier than the normal scenario in unialgal cultures. Most of them peaked earlier because of faster growth in the HT treatment, although some of the peaks were not significant. In the field environment, the earlier peaks were not only caused by increasing temperature, but also by improvements in the turbulence and light conditions [61].

### 4.2. The Effect of Interspecific Interactions on the Response of Diatoms to Warming

The concentration of dissolved silicate showed a rapid declining trend in the first 10 days in all cultures (Figure S2), and most diatom species had lower abundance in mixed cultures than in unialgal cultures from the 10th day, except for *F. nanana* (Figure 6). Nutrients competition might be the main interaction among these algae species during the phase of nutrient decline. The competition for nutrients led to relatively less available resource and lower abundance for diatom species, even if the species (*A. catenatum* and *U. ulna*) should have higher abundance in a warming environment. Subsequently, nutrient competition might be alleviated when the concentration of silicate showed a rising trend (Figure S2), thus, the limitations of light and space likely became the main factors for restricting the growth of diatoms. The high biovolume of algae caused self-shading in mixed cultures and led to the species with smaller biomass receiving less light energy. The size and shape of algae may be important factors affecting their ability to compete for space [64]. The small size of *A. catenatum* means that it occupies a relatively small space when its individual numbers increase, while the large size of unicellular *U. ulna*, tubular-shaped *A. ambigua*, and especially stellate-shaped *A. formosa* results in them having large spatial requirements for proliferation. Harmful allelochemicals produced by freshwater diatoms have not been fully understood, and whether there is any allelopathy between the diatom species used in our experiments requires further research. In this study, interspecific interactions in mixed cultures weakened the intensity of the responses of *A. catenatum* and *U. ulna* to warming, and even changed the pattern of the response to warming by *A. formosa* (Figure 6). Although, the specific interactive processes among these species need to be further determined, we can now infer that those interspecific interactions affected the specific responses of diatoms to warming scenarios.

Both theoretical and empirical studies have suggested that interspecific interactions could substantially alter the responses of species to climate change [65,66]. In the present study, interspecific interactions affected the peak growth of diatoms in co-cultures. The elevated temperature did not advance the growth peaks of *A. ambigua* and *A. formosa* in co-cultures, probably because they were more affected by interspecific interactions than by the elevated temperature. In co-cultures, the growth of *U. ulna* and *F. nanana* peaked much earlier in the warming scenarios (Figure 6). It is possible that the growth of coexisting diatoms was limited by space much earlier in the warming scenarios.

Our results reinforce the findings of some recent studies that support an important role of interspecific interactions in species responses to warming. For example, Lin and Morin (2004) found that the competition for food between *Colpidium* and *Paramecium* makes unexpected responses of *Paramecium* to warming [36]. Singer et al. (2013) suggested that interspecific interactions between a host and an obligate species affected species responses to climate shifts [67]. This present study builds on the results of those previous studies and suggests that interspecific interactions could also play important roles in algal species responses to warming.

## 5. Conclusions

Our results indicated that diatoms with an obvious seasonal succession adapt to different optimal temperature ranges, and their specific responses to warming were expected if there were no interspecific interactions. This will likely lead to an increased abundance of warm-adapted species in spring and cold-adapted species in early spring, with no significant effect on eurythermal species, and an advance in the timing of the peak growth of diatoms. However, mixed culture systems demonstrated that the responses of diatoms to simulated future spring warming were affected by interspecific interactions, in both the abundance and the timing of their growth peaks. Furthermore, our experimental system is far simpler than most natural communities, with only five diatom species and without predators. In natural environments, interspecific interactions are inevitable, and it is therefore extremely difficult to accurately predict how climate warming will affect species and communities because of the complex relationships among various organisms. This important issue cannot be ignored in studies of the response to climate warming.

## Figures and Tables

**Table 1:** Micrographs, size, isolation season, and the range of growth temperature in the field of the five diatom species. The range of growth temperature was obtained from a generalized additive model (GAM) which analyzed the relationship between the abundance of diatom species and water temperature [41].

Diatom Species	Micrographs	Volume (μm^3^·cell^−1^)	Isolation Season	Range of Growth Temperature in the Field
*Achnanthidium catenatum*	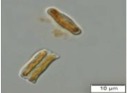	65.2	Autumn	15–28 °C
*Fragilaria nanana*	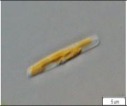	374.5	Autumn	10–30 °C
*Ulnaria ulna*	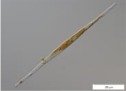	3169.0	Summer	11–33 °C
*Asterionella formosa*	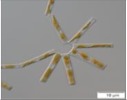	625.5	Winter	6–15 °C
*Aulacoseira ambigua*	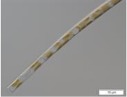	331.0	Autumn	6–20 °C

**Table 2:** Hourly temperature changes every day in the two settings of the fluctuant temperatures. LT: low temperature, HT: high temperature (+4 °C).

O’clock	Day 1		Day 42		Day 43		Day 50	
	LT (°C)	HT (°C)	LT (°C)	HT (°C)	LT (°C)	HT (°C)	LT (°C)	HT (°C)
9:00	10	14	30	34	31	-	34	-
11:00	12	16	32	36	33	-	36	-
13:00	14	18	34	38	35	-	38	-
15:00	13	17	33	37	34	-	37	-
17:00	12	16	32	36	33	-	36	-
19:00	11	15	31	35	32	-	35	-
21:00	10	14	30	34	31	-	34	-
23:00	9	13	29	33	30	-	33	-
1:00	8	12	28	32	29	-	32	-
3:00	7	11	27	31	28	-	31	-
5:00	6	10	26	30	27	-	30	-
7:00	8	12	28	32	29	-	32	-

## Supplementary Materials

The following are available online at http://www.mdpi.com/2076-2607/6/3/82/s1, Figure S1: Seasonal variation of the five dominant diatom species (*U. ulna* and *F. nanana* presented two peaks in abundance in a year, and the black bar and gray bar represented peak of abundance formed at different periods). Figure S2: The concentration of dissolved silicate in mixed cultures and unialgal cultures. (A) Mixed culture; (B–F) Unialgal culture; (B) *Achnanthidium catenatum*; (C) *Fragilaria nanana*; (D) *Ulnaria ulna*; (E) *Asterionella formosa*; (F) *Aulacoseira ambigua*.
