# Supplementary figures and images for: The Species-Specific Responses of Freshwater Diatoms to Elevated Temperatures Are Affected by Interspecific Interactions
Source: Microorganisms. 2018 Aug 7;6(3):82. doi: 10.3390/microorganisms6030082 (PMC6163879; doi:10.3390/microorganisms6030082)

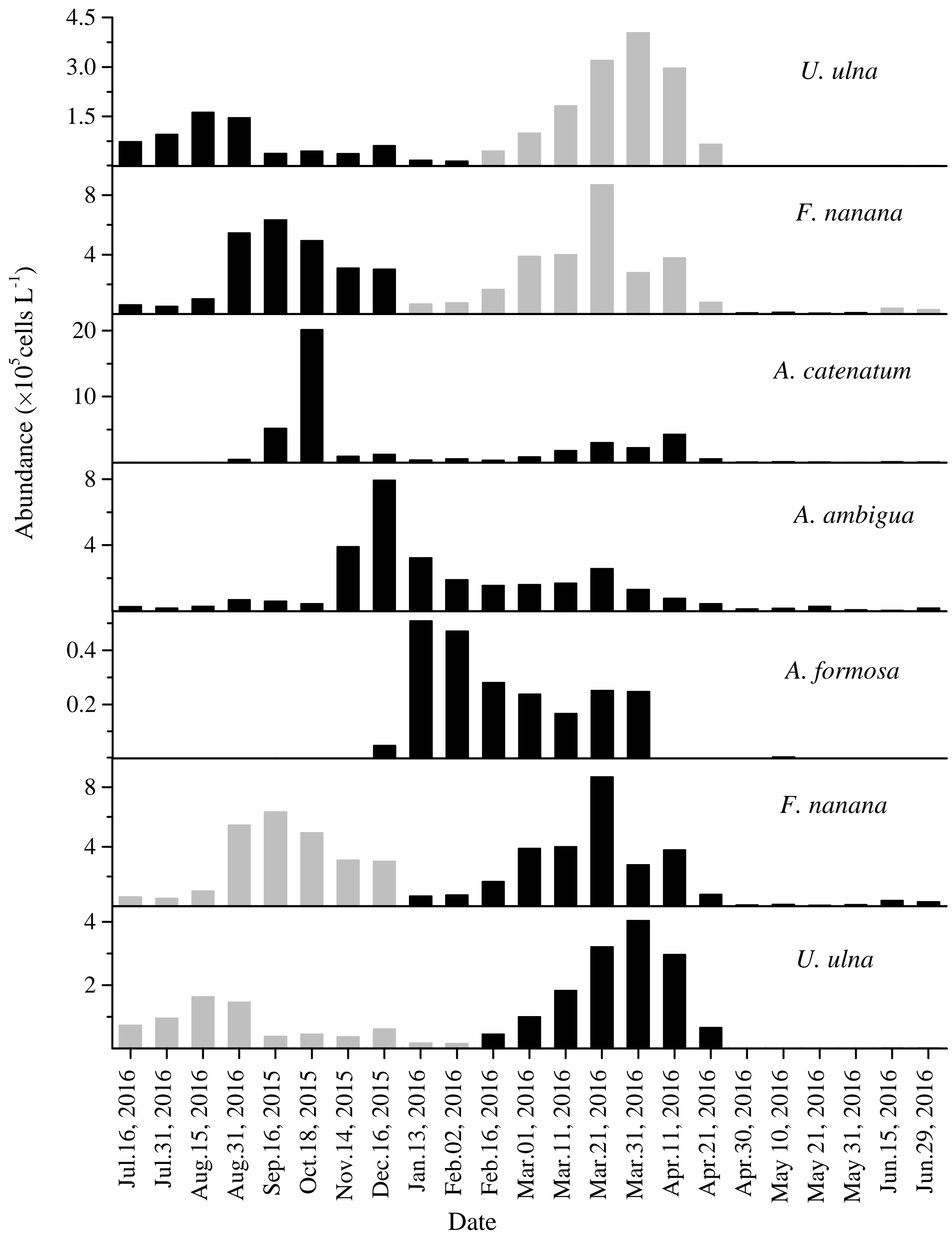

Supplement: Supplementary file 1 [file microorganisms-06-00082-s001.zip › Figure S1.pdf]
