# Supplementary material for: The Species-Specific Responses of Freshwater Diatoms to Elevated Temperatures Are Affected by Interspecific Interactions
Source: Microorganisms. 2018 Aug 7;6(3):82. doi: 10.3390/microorganisms6030082 (PMC6163879; doi:10.3390/microorganisms6030082)
Supplement: Supplementary file 1 [file Figure S2.pdf › Figure S2.pdf]

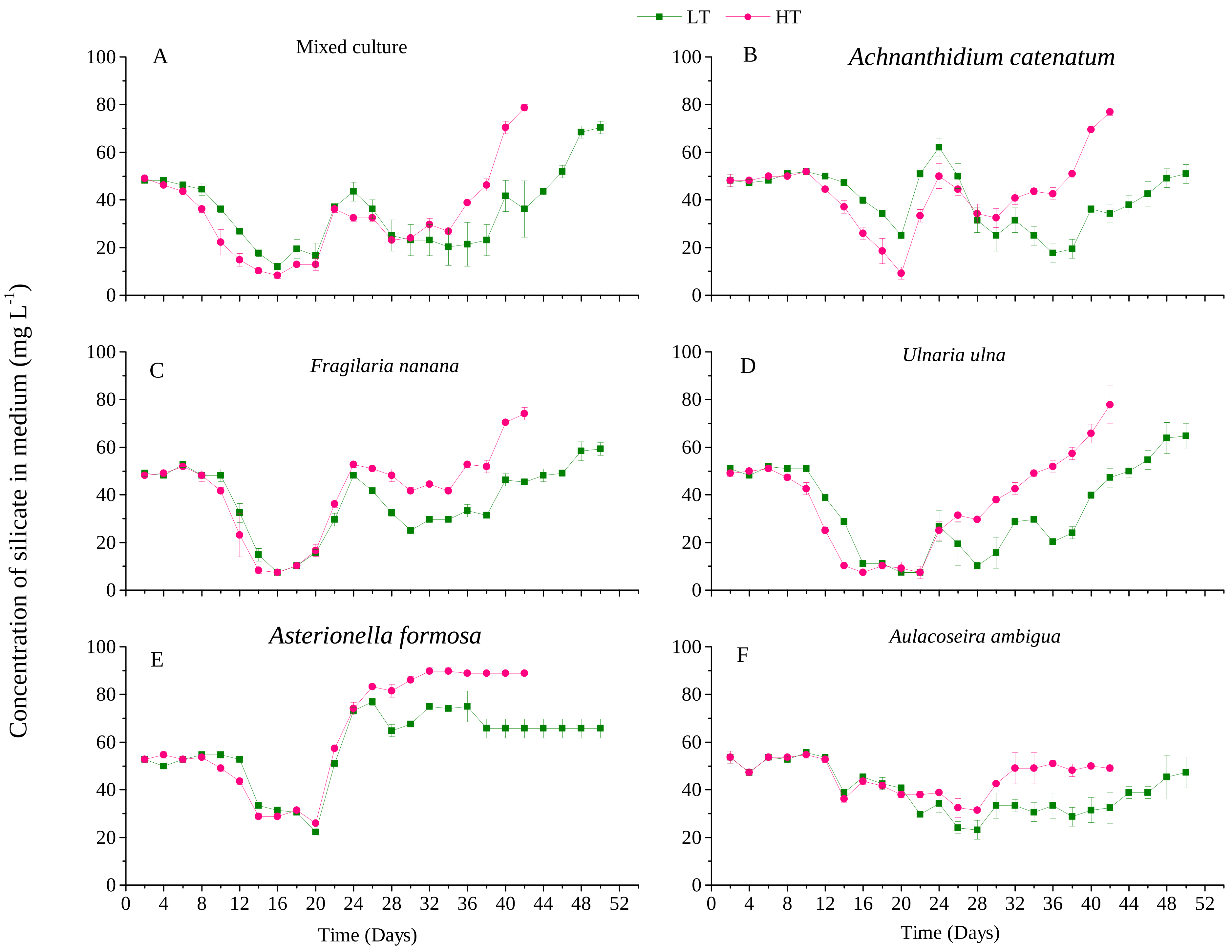

LT
HT
Concentration of silicate in medium (mg L-1)
A
Mixed culture
B
Achnanthidium catenatum
C
Fragilaria nanana
D
Ulnaria ulna
E
Asterionella formosa
F
Aulacoseira ambigua
0
20
40
60
80
100
0 4 8 12 16 20 24 28 32 36 40 44 48 52
Time (Days)
Time (Days)
